# Supplementary material for: Residues and Safety Assessment of Cyantraniliprole and Indoxacarb in Wild Garlic (Allium vineale)
Source: Toxics. 2023 Feb 25;11(3):219. doi: 10.3390/toxics11030219 (PMC10056846; doi:10.3390/toxics11030219)
Supplement: Supplementary file 1 [file toxics-11-00219-s001.zip › toxics-2153035-supplementary.pdf]

toxics

MDPI

Supplementary Materials: Residues and Safety Assessment of Cyantraniliprole and Indoxacarb in Wild Garlic (*Allium vineale*)

Syed Wasim Sardar, Jeong Yoon Choi, Yeong Ju Jo, Abd Elaziz Sulieman Ahmed Ishag, Min-woo Kim and Hun Ju Ham

Table S1. Physicochemical properties of cyantraniliprole and indoxacarb in wild garlic.

	Cyantraniliprole	Indoxacarb
Chemical structure	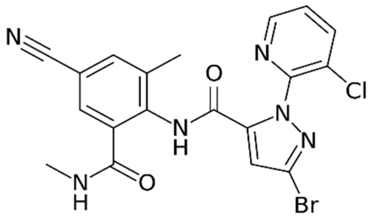	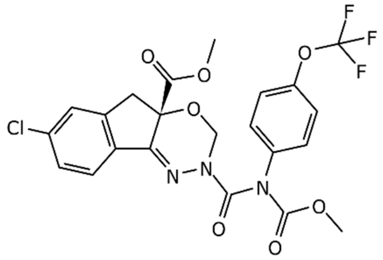
IUPAC name	5-bromo-2-(3-chloropyridin-2-yl)-*N*-[4-cyano-2-methyl-6-(methylcarbamoyl)phenyl]pyrazole-3-carboxamide	methyl (4*aS*)-7-chloro-2-[methoxycarbonyl-[4-(trifluoromethoxy)phenyl]carbamoyl]-3,5-dihydroindeno[1,2-e][1,3,4]oxadiazine-4*a*-carboxylate
Mol. wt.	473.7	527.8
K_{ow} logP	3.2	4.65

Table S2. Application dose of cyantraniliprole and indoxacarb.

Crop	Pesticides	Formulation type	A.I.[1)](%)	Application times	Pre-harvest application intervals	Dilution
Wild garlic	Cyantraniliprole	SC[2)]	5%	2	14-0	2,000
	Indoxacarb	WP[3)]	10%	2	14-0	1,000

Table S3. LC-MS/MS operating conditions for the analysis of cyantraniliprole in wild garlic.

HPLC	Dionex Ultimate 3000 (Thermo Science, USA)			
Column	Imtakt Unison UK-C18 (2.0 mm I.D.×100 mm×3.0 μm)			
Column Temp.	40℃			
	Flow(mL/min.)	Time	A (%)	B (%)
Gradient condition	0.3	0.0	95.0	5.0
		1.0	95.0	5.0
		1.5	5.0	95.0
		5.0	5.0	95.0
		7.5	95.0	5.0
		10.0	95.0	5.0
**MS/MS**	**TSQ Quantum Access Max (Thermo Science, USA)**			
Ionization mode	ESI+			
Spray voltage	4,000 V			

Capillary Temp.	320℃
Vaporizer Temp.	350°C
Sheath Gas Pressure (N2)	40 units
Aux Gas Pressure (N2)	15 units
Collision gas & pressure	Argon, 1.5 Torr
Scan event	SRM (Selected Reaction Monitoring) mode

Pesticide	Precursor ion	Product ion	CE	Retention time (min.)
Cyantraniliprole	475.012	285.908	17	5.86
		444.019	19	

Table S4. LC-MS/MS operating conditions for the analysis of indoxacarb in wild garlic.

**HPLC**	**Dionex Ultimate 3000 (Thermo Science, USA)**			
Column	Unison UK-C18 (2.0 mm I.D.×100 mm×3.0 μm)			
Column Temp.	40℃			
Gradient condition	Flow(mL/min.)	Time	A (%)	B (%)
	0.35	0.0	95.0	5.0
		1.0	95.0	5.0
		1.5	5.0	95.0
		6.0	5.0	95.0
		6.5	95.0	5.0
		8.0	95.0	5.0
		10.0	95.0	5.0

**MS/MS**	**TSQ Quantum Access Max (Thermo Science, USA)**
Ionization mode	ESI+
Spray voltage	3,500 V
Capillary Temp.	200℃
Vaporizer Temp.	220℃
Sheath Gas Pressure (N2)	20 units
Aux Gas Pressure (N2)	10 units
Collision gas & pressure	Argon, 1.5 Torr
Scan event	SRM (Selected Reaction Monitoring) mode

Pesticide	Precursor ion	Product ion	CE	Retention time (min.)
Indoxacarb	528.400	150.258	27	6.01
		203.150	36	

Table S5. Linearity of calibration curve used for the quantification of cyantraniliprole and indoxacarb.

**Matrix**	**Pesticides**	**Equation**	**R^2**
Wild Garlic	Cyantraniliprole	y = 467481.3298x - 2123.347728	0.994
	Indoxacarb	y = 1566198.513x - 236.7621929	0.993

Table S6. Recovery of cyantraniliprole and indoxacarb from wild garlic.

**Crops**	**Pesticides**	**Fortification level (mg/kg)**	**Recoveries (%)**		
			***Rep 1**	**Rep 2**	**Rep 3**
Wild garlic	Cyantraniliprole	0.01	91.2	101.0	90.4
		0.1	95.1	105.7	95.3
	Indoxacarb	0.01	116.1	108.1	110.1
		0.1	104.2	107.0	103.6

*Repetition.

Table S7. Residual amount of cyantraniliprole and indoxacarb.

Crops	Pesticides	Spray days before harvest	Residue amount (mg/kg)		
			*Rep 1	Rep 2	Rep 3
Wild garlic	Cyantraniliprole	Control	<0.01	<0.01	<0.01
		0	0.036	0.035	0.034
		3	0.031	0.028	0.030
		7	0.09	0.01	0.01
		14	<0.01	<0.01	<0.01
	Indoxacarb	Control	<0.01	<0.01	<0.01
		0	0.147	0.136	0.137
		3	0.020	0.022	0.019
		7	0.014	0.012	0.017
		14	<0.01	<0.01	<0.01

*Repetition.

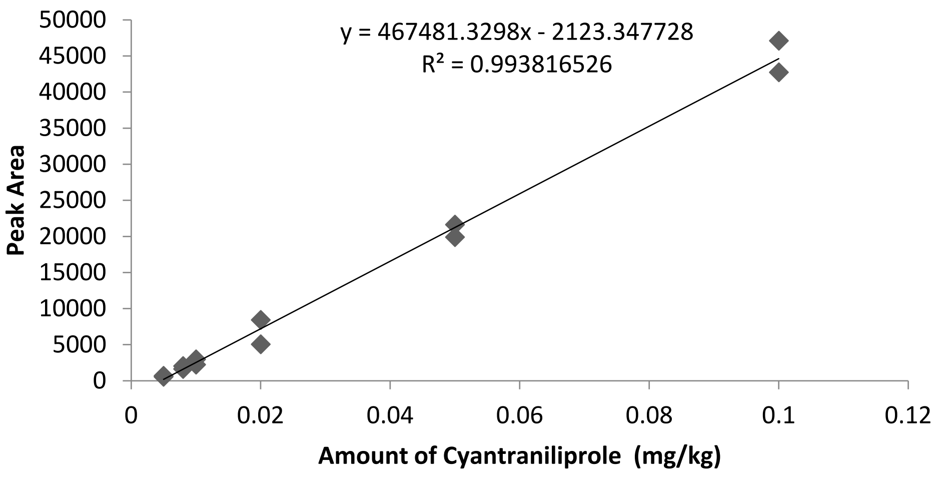

Figure S1. Calibration curve used for quantification of cyantraniliprole in wild garlic.

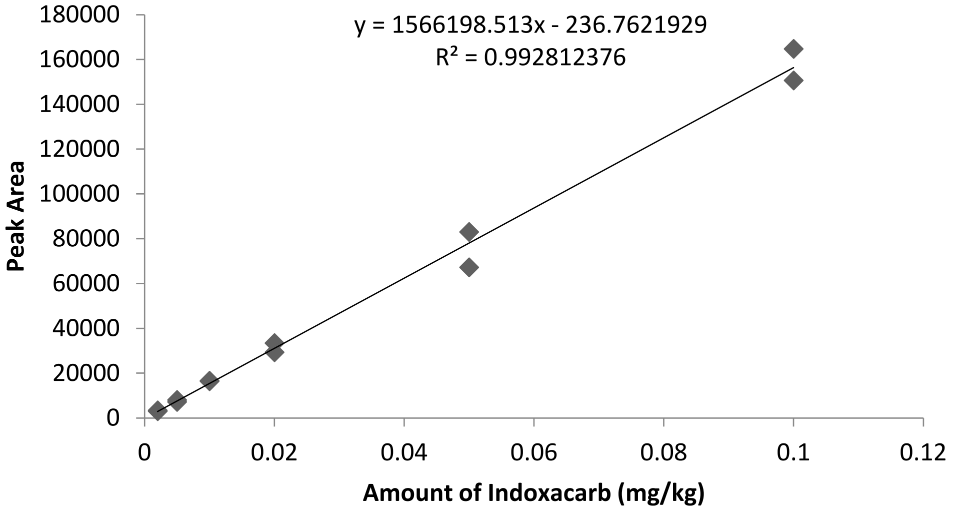

Figure S2. Calibration curve used for quantification of indoxacarb in wild garlic.
